# LipidSeq: a next-generation clinical resequencing panel for monogenic dyslipidemias[S]

**DOI:** 10.1194/jlr.D045963

**Published:** 2014-04

**Authors:** Christopher T. Johansen, Joseph B. Dubé, Melissa N. Loyzer, Austin MacDonald, David E. Carter, Adam D. McIntyre, Henian Cao, Jian Wang, John F. Robinson, Robert A. Hegele

**Affiliations:** Robarts Research Institute, Schulich School of Medicine and Dentistry, Western University, London, Ontario, Canada N6A 5B7

**Keywords:** next generation sequencing, DNA diagnosis, familial dyslipidemia, Sanger sequencing, mutations, genetic risk score, polygenic dyslipidemia

## Abstract

We report the design of a targeted resequencing panel for monogenic dyslipidemias, LipidSeq, for the purpose of replacing Sanger sequencing in the clinical detection of dyslipidemia-causing variants. We also evaluate the performance of the LipidSeq approach versus Sanger sequencing in 84 patients with a range of phenotypes including extreme blood lipid concentrations as well as additional dyslipidemias and related metabolic disorders. The panel performs well, with high concordance (95.2%) in samples with known mutations based on Sanger sequencing and a high detection rate (57.9%) of mutations likely to be causative for disease in samples not previously sequenced. Clinical implementation of LipidSeq has the potential to aid in the molecular diagnosis of patients with monogenic dyslipidemias with a high degree of speed and accuracy and at lower cost than either Sanger sequencing or whole exome sequencing. Furthermore, LipidSeq will help to provide a more focused picture of monogenic and polygenic contributors that underlie dyslipidemia while excluding the discovery of incidental pathogenic clinically actionable variants in nonmetabolism-related genes, such as oncogenes, that would otherwise be identified by a whole exome approach, thus minimizing potential ethical issues.

Next generation sequencing (NGS) broadly refers to the new wave of DNA sequencing technologies that have emerged in the post-Sanger sequencing era (1, 2). NGS platforms provide massively parallel sequencing of millions of DNA fragments, which enables the rapid sequencing of whole genomes in less than a day and at a fraction of the cost compared with Sanger sequencing. The recent clinical application of NGS has revolutionized the ability to rapidly develop molecular diagnoses in inherited disease (3, 4), especially monogenic diseases (2, 5, 6). Furthermore, the cost of NGS is rapidly decreasing, and has made tangible the prospect of incorporating genome-based diagnosis into medical care. In this regard, the US Food and Drug Administration recently approved Illumina's MiSeqDx for NGS applications in the clinical setting (7). These developments are relevant for genetic dyslipidemias, as the comprehensive detection of genome-wide variation opens up new approaches to further characterize the polygenic basis of complex metabolic traits (8, 9).

Clinically, the identification of causative genetic mutations in patients with suspected familial hypercholesterolemia (FH) is a criterion for diagnosing “definite FH” or “probable FH” in two widely used clinical algorithms (10, 11). Also, screening for causative mutations in candidate genes in lipolysis for the diagnosis of familial chylomicronemia is supplanting traditional biochemical diagnostic methods, such as LPL activity in plasma collected postheparin infusion (12, 13). It is not presently clear whether molecular diagnosis will be clinically important for the >20 other monogenic disorders of lipid and lipoprotein metabolism whose molecular basis has been solved (11), particularly as feasibility and efficiency via Sanger sequencing have limited the broad integration of clinical resequencing for dyslipidemia patients. However, NGS now presents a tool to evaluate the effectiveness of genome-based diagnosis in monogenic dyslipidemias and address clinical feasibility and applicability, as well as potential ethical concerns that come with genome-wide variant detection.

Here, we have designed and evaluated a targeted resequencing panel for monogenic dyslipidemias termed LipidSeq. Our objective was to utilize an NGS-based approach to facilitate molecular diagnosis of dyslipidemias in patient samples studied at the Blackburn Cardiovascular Genetics Laboratory with the intention of replacing existing Sanger sequencing-based methods. Our laboratory performs molecular diagnosis of largely clinical samples from patients covering a range of dyslipidemias that are characterized by: *1*) very high levels of LDL cholesterol, including FH and related conditions; *2*) very low levels of LDL cholesterol, including abetalipoproteinemia and hypobetalipoproteinemia (HBL); *3*) very high levels of HDL cholesterol; *4*) very low levels of HDL cholesterol, including Tangier disease and familial deficiencies of apoA-1 and LCAT; and *5*) very high levels of TG, including familial chylomicronemia (14). The laboratory also receives samples for molecular diagnosis of miscellaneous dyslipidemias, as well as monogenic forms of diabetes, such as lipodystrophy syndromes (15, 16), and mature onset diabetes of the young (MODY) (17). Our aims were to: *1*) determine the accuracy of NGS compared with traditional Sanger sequencing with respect to variant discovery in monogenic dyslipidemias and related metabolic disorders; *2*) evaluate the reproducibility of variant discovery between samples; and *3*) assess the potential diagnostic utility of targeted high-throughput sequencing technology in the clinic.

## METHODS

### Study design

#### Sequence capture.

We used the Nextera Custom Enrichment kit (Illumina, San Diego, CA) to capture genomic regions corresponding to 73 genes (supplementary Table I) and 178 SNPs (supplementary Table II) associated with clinical dyslipidemias and related metabolic disorders, comprising 689 kb of sequence per sample. Some content was also included for experimental research purposes. Each exon of every coding isoform of the 73 targeted genes was captured, as well as 150 bp pads into the introns and an extra 2 kb of upstream sequence and 500 bp of downstream sequence. We also included SNPs that were chosen based on their contribution to polygenic risk scores for LDL and HDL cholesterol and TG, based on genome-wide association studies (GWASs) of lipid traits (18). SNPs were captured using a single probe centered on the variant of interest. Chromosome scaffold coordinates were obtained from the University of California Santa Cruz genome browser using the February 2009 GRCh37/hg19 genome build (19) and were submitted to the Illumina Online Design Studio (Illumina, San Diego, CA).

#### Sample selection.

In total, 84 patient samples were sequenced in this study. The validation portion of the study included 24 different hypertriglyceridemia (HTG) patients whose genomic DNA was sequenced extensively in the course of previous unrelated projects involving Sanger sequencing (20, 21). The technical replication portion of the study included 12 samples that were duplicate HTG samples, including 6 genomic DNA samples and 6 whole genome amplified (WGA) DNA samples. The remaining 48 samples were obtained from patients with a range of phenotypes, including 19 referred with possible FH (but without any prior molecular data), 10 who were FH patients with no mutations in the candidate FH genes (*LDLR*, *APOB*, and *PCSK9*), 14 who were recently diagnosed with severe HTG, and 5 who were referred with other rare monogenic phenotypes ascertained clinically, including 2 with familial partial lipodystrophy (FPLD), 2 with MODY, and 1 with HBL. FH patients were diagnosed as probable FH based on the Dutch Lipid Clinic Criteria prior to assessment with LipidSeq (11). HTG patients included in this study had fasting plasma TG >10 mmol/l. All patients provided informed consent under a protocol approved by the Research Ethics Board at Western University (#07920E).

### Sequencing

#### Sample preparation.

DNA was processed in batches of 12 samples. The initial quality and quantity of genomic DNA samples was assessed by visualization on a 1% agarose gel and using a NanoDrop 1000 spectrophotometer (Thermo Fisher Scientific, Inc., Waltham, MA). The DNA was then diluted to a starting concentration of 3.0 ± 0.5 ng/μl and measured using a Qubit 2.0 fluorometer (Invitrogen, Carlsbad, CA). WGA DNA samples were diluted to a starting concentration of 3.0 ± 0.5 ng/μl using the Qubit 2.0 fluorometer.

Library preparation was conducted at the London Regional Genomics Centre following the Nextera Custom Enrichment Sample Preparation Guide. Briefly, samples were enzymatically fragmented, PCR amplified with individual sample barcodes, equimolar pooled, hybridized to the custom designed target probes (two cycles of 18 h each with multiple washes), and PCR amplified again to select the final target sequence. All steps were conducted in accordance with the manufacturer's recommendations. DNA pull-down steps used the Magnetic Stand-96 (Life Technologies, Gaithersburg, MD) and PCR amplification used the Veriti thermocycler (Applied Biosystems, Foster City, CA). Mid-preparation sample quality was verified using the Agilent 2100 BioAnalyzer (Agilent Technologies, Palo Alto, CA); final libraries were quantified using the KAPA quantitative PCR library quantification kit (KAPA Biosystems, Woburn, MA) on a ViiA 7 real-time PCR system (Life Technologies).

#### Sequencing parameters.

Indexed samples were pooled in equimolar ratios of 500 ng. Once combined, 16 pM of denatured pooled library was loaded on to a standard flow-cell on the Illumina MiSeq personal sequencer (Illumina, San Diego, CA) using 2 × 150 bp paired-end chemistry according to the manufacturer's instructions. PhiX (1%) was spiked in as a positive control for sequencer performance. Sequencing quality control was assessed using multiple parameters in Illumina MiSeq Reporter and visualized either in Illumina BaseSpace or locally using Illumina sequencing analysis viewer. Demultiplexed FASTQ files were downloaded for each sample and processed individually using downstream software packages, as described below.

### Variant discovery and annotation

#### Sequence alignment and variation discovery.

Sequence alignment and variant calling were conducted using a custom automated workflow designed in CLC Bio Genomics Workbench v6.5 β (CLC Bio, Aarhus, Denmark). First, sequencing reads were imported from FASTQ files and mapped to the full human genome GRCh37/hg19 build. Second, default settings of two built-in protocols were used to improve read mapping including a local realignment to improve mapping around insertion-deletion mutations (indels) and removal of PCR duplicates. Finally, a quality-based variant detection tool was used to call sequence variants according to a minimum 10-fold coverage and 20% read frequency. These parameters were chosen to minimize false negatives by facilitating identification of variants in low-coverage regions for follow-up using Sanger sequencing if necessary. Target region summary statistics were generated in tab-delimited format and variant reports for each sample were exported in VCF format for use in downstream annotation pipelines. Only variants identified within 10 bp of target regions were included in these analyses.

#### Variant annotation.

Variant annotation was performed using ANNOVAR (22). All genome coordinates and database files made reference to human genome build GRCh37/hg19. Several databases were downloaded from ANNOVAR to facilitate annotation, including RefSeq (February 2013 update), dbSNP137 (July 2013; nonflagged variants), the National Heart, Lung, and Blood Institute Exome Sequencing Project (ESP; June 2012 update), 1000 Genomes Project (G1K; February 2012 update), and sorting intolerant from tolerant (SIFT) and PolyPhen-2 databases (May 2011 updates). Variants were also compared against the Human Gene Mutation Database using an automated script.

Variants were classified into four functional categories: *1*) coding variants found in translated portions of exons; *2*) clear splicing variants found within two nucleotides of an intron-exon boundary; *3*) untranslated region (UTR) variants found in the 5′ or 3′ UTR; or *4*) noncoding variants found in introns with no effect on splicing or intergenic variants. Single nucleotide variants were classified as nonsynonymous (missense and nonsense) or synonymous variants, whereas indels were classified as insertions or deletions either causing a frameshift or not (i.e., in-frame). Novel variants were defined as having no frequency listed in either ESP or G1K, and no rsID in dbSNP. Among novel variants, three were identified in >10% of study samples and were deemed sequencing artifacts; these variants were not included in summary statistics.

### Statistical analyses

All statistical analyses were conducted in SAS v9.2 (Cary, NC). Data are expressed as mean ± SD. Comparisons between means were conducted using unpaired *t*-test assuming unequal variances. Statistical significance was defined as a two-tailed *P* value <0.05.

## RESULTS

### Validation

#### Sequencing quality.

Two validation runs of 12 samples produced comparably high quality sequence data as measured by several quality control filters (**Table 1**). Run 1 produced a total of ∼15,000,000 reads of which ∼13,000,000 (86.1%) passed standard instrument quality control filters. From these reads, 96% of indexes were correctly deciphered and 93% of reads exceeded a Phred quality score of Q30, signifying an error probability <1/1,000. Run 2 produced a total of ∼20,000,000 reads of which ∼17,000,000 (84.9%) passed standard instrument quality control filters. From these reads, 96% of indexes were correctly deciphered and 93% of reads exceeded a Phred quality score of Q30.

**TABLE 1.** Quality control measures for sequencing validation runs

Run Parameter	Validation Run 1	Validation Run 2	Validation Run Mean
Cluster density (×10^3^/mm^2^)	966	1165	1065
Total reads (×10^3^)	14,915	19,515	17,215
Reads PF (×10^3^)	12,847	16,583	14,715
Reads PF (%)	86.1	84.9	85.5
Reads Identified*^a^* (%)	96.0	95.7	95.9
Reads > Q30*^a^* (%)	92.7	92.7	92.7
Mean coverage (±SD)	298.1 (±88.6)	392.2 (±48.1)	345.1 (±84.67)
Targets with mean coverage <30× (%)	2.6 (±1.0)	1.8 (±0.4)	2.2 (±0.9)
Targets with min coverage <30× (%)	6.8 (±2.2)	4.9 (±0.4)	5.8 (±1.8)
Targets with max coverage <30× (%)	1.9 (±0.9)	1.3 (±0.3)	1.6 (±0.7)

Validation runs included 24 independent samples with 12 samples per run. PF, passed filter.

a Percentage of reads identified and percentage of reads >Q30 are based on reads SD.

Each sample index was fairly well represented on the flow cell. The mean (±SD) proportion of reads identified per sample was 8.0% (±2.5%) in run 1 and 8.0% (±1.0%) in run 2, as would be expected (100%/12 samples = 8.3%/sample). There was greater variation among samples within run 1: two samples had a proportion of total reads of ∼4.0%, whereas another two samples had a proportion of total reads of ∼11.0%. Samples within run 1 had proportions of index reads between 6.0 and 10.0%. Results for all samples in both runs were within 2 SDs of the mean.

Our sequence capture reagents were designed to exceed 100-fold (100×) coverage of 1,043 target sites representing 689,400 bp (Table 1). This approach resulted in a mean (±SD) read depth per base pair of 298.1 (±88.6) in run 1 and 392.1 (±48.1) in run 2. The percentage of targets with a mean coverage <30× was 2.6% (±1.0%) in run 1 and 1.8% (±0.4%) in run 2, whereas the percentage of these with a minimum coverage <30× was relatively higher, encompassing 6.8% (±2.2%) of run 1 targets and 4.5% (±0.4%) of run 2 targets. Variant detection was conducted at all locations where read depth was >20× coverage. Conversely, targets that failed in their entirety based on a maximum coverage <30× only occurred in 1.9% (±0.9%) and 1.3% (±0.3%) of targets in runs 1 and 2, respectively. These statistics were all superior for run 2 given the greater significant increase in reads captured and sequenced. A higher resolution investigation of targets with poor coverage revealed that the proportion of individual base pairs with coverage <30× was only 2.5% (±0.8%) in run 1 and 1.9% (±0.3%) in run 2, and a significant proportion of these sites were base pairs with 0× coverage, including 0.5% (±0.1%) of base pairs in run 1 and 0.6% (±0.1%) in run 2.

#### Technical concordance between samples and DNA inputs.

We conducted additional sequencing to assess the technical concordance between samples. For six samples, we selected one technical replicate prepared from genomic DNA and one prepared from WGA DNA. The NGS run containing these 12 samples performed exceptionally well, producing a total of ∼21,000,000 reads of which ∼16,500,000 (79.2%) passed standard instrument quality control filters. From these reads, 97% of indexes were correctly deciphered and 90% of reads exceeded a Phred quality score of Q30.

Interestingly, sample performance indexes were significantly higher among genomic versus WGA DNA samples, with mean (±SD) of 10.3% (±1.4%) versus 5.8% (±1.5%) reads per sample (*P* = 0.0004; **Table 2**), respectively. The greatest proportion of indexes for a single technical replicate was 11.9% corresponding to genomic DNA sample input, whereas the lowest proportion of indexes was 4.2% corresponding to a WGA DNA sample input. Sequencing coverage was also significantly better among genomic versus WGA DNA samples (Table 2). For instance, the mean (±SD) read depth per base in genomic DNA versus WGA DNA samples was 399.4 (±78.2) versus 264.5 (±58.0), respectively (*P* = 0.008); all other measures of coverage were better among genomic DNA sample inputs.

**TABLE 2.** Quality control measures compared between genomic and WGA DNA sample inputs

Run Parameter	Genomic DNA (n = 6)	WGA DNA (n = 6)	*P*
Mean index reads (%) (±SD)	10.3 (±1.44)	5.84 (±1.50)	0.0004
Mean coverage (±SD)	399.4 (±78.2)	264.5 (±58.0)	0.008
Targets with mean coverage <30× (%)	1.0 (±0.09)	4.0 (±1.5)	0.005
Targets with min coverage <30× (%)	4.8 (±0.8)	9.9 (±2.7)	0.005
Targets with max coverage <30× (%)	0.6 (±0.1)	2.6 (±1.1)	0.007

Concordance rates were excellent in both categories of technical replicates with an overall concordance rate of 95.2%; however, genomic DNA replicates performed with significantly higher concordance compared with WGA replicates (**Table 3**). The number of concordant calls was no different among genomic versus WGA DNA samples (464.5 vs. 453.3, respectively; *P* = 0.48), however the number of discordant calls was lower among genomic versus WGA DNA samples (18.0 vs. 28.6, respectively; *P* = 0.01). This difference was driven by variants that were exclusively called in either the original or replicate sample, rather than variants that were identified in both samples but called with different genotypes (supplementary Table III). Furthermore, the vast majority of discordant variants were found in regions of lower clinical importance including the 5′ UTRs or noncoding regions. These regions provide limited diagnostic value as their impact on protein structure and function is less clear in comparison to nonsynonymous variants. The overall concordance rate was 96% among genomic DNA samples and 94% among WGA DNA samples (*P* = 0.008). These findings demonstrate that technical concordance among genomic DNA is excellent according to multiple quality control metrics, although WGA input DNA is sufficient for sequencing and the identification of clinically relevant variants.

**TABLE 3.** Concordance of variants identified in technical replicates separated by location

		Concordant Calls*^a^*				Discordant Calls*^b^*			
Sample ID	Input DNA	Exon: Coding	Exon: UTR	Noncoding	Total	Exon: Coding	Exon: UTR	Noncoding	Total
3344	Genomic	89	135	245	469	1	9	6	16
	WGA	86	134	240	460	4	13	11	28
3645	Genomic	100	135	236	471	0	12	8	20
	WGA	100	126	235	461	0	19	11	30
3732	Genomic	96	160	245	501	0	12	6	18
	WGA	93	151	238	482	3	20	14	37
4221	Genomic	98	132	247	477	0	6	9	15
	WGA	96	130	247	473	2	10	6	18
4270	Genomic	83	122	221	426	0	9	8	17
	WGA	81	117	219	417	2	14	9	25
4667	Genomic	93	124	226	443	2	8	12	22
	WGA	89	114	224	427	5	18	11	34

Noncoding refers to intronic and intergenic variants.

a Concordant calls refer to a variant being identified and identical genotype called between the original and replicate sample.

b Discordant calls refer to a variant identified in both samples but genotype was called differently, or the variant was called in either the original or replicate sample.

#### Validating LipidSeq variant detection with Sanger sequencing.

The two validation runs were composed of 24 unique samples that were previously extensively Sanger sequenced in unrelated projects using the ABI 3730 automated sequencer Sanger sequencing protocol (8). Concordance was compared for both common and rare variants found in translated sequences from 18 HTG subjects sequenced across eight genes involved in HTG susceptibility (including >17.2 kb per person) and six additional subjects with monogenic phenotypes were sequenced across three to nine genes (including 6.3 kb to 27.5 kb per person). Among 18 polygenic HTG patients, or HTG patients with no candidate HTG mutations (23), Sanger sequencing identified 32 distinct sequence variants including 19 nonsynonymous variants and 13 synonymous variants, corresponding to a total of 114 alternate genotypes within these samples. All 114 alternate genotypes were identified by the MiSeq protocol, including 6 alternate genotypes that were initially missed or misannotated by Sanger sequencing, and correctly identified by the MiSeq upon Sanger sequence confirmation. Additionally, we compared Illumina-determined genotypes for HTG samples for a subset of 44 SNPs selected from GWASs of plasma lipids (13), which had been previously genotyped in our lab using TaqMan reagents. Two SNPs consistently failed to be captured, including the fatty acid desaturase 1/2/3 (*FADS1/2/3*) gene cluster rs174547 and cholesteryl ester transfer protein (*CETP*) rs173539, although these SNPs also consistently failed in our TaqMan genotyping assays, suggesting that local DNA properties may interfere with attempts to genotype these loci. Within 18 samples at the remaining 42 genotypes, we achieved 100% concordance for variants being queried.

Among samples from patients referred with potential monogenic phenotypes, Sanger sequencing identified 42 distinct sequence variants, including 24 nonsynonymous variants and 18 synonymous variants, corresponding to 68 alternate genotypes. Again, all 68 alternate genotypes were detected by the MiSeq. Therefore, among all validation samples, 182 nonreference genotypes were correctly called by the MiSeq and 396,241 reference genotypes were correctly called by the MiSeq using Sanger sequencing as the comparator. Such findings demonstrate the high accuracy afforded by the MiSeq protocol to detect clinically relevant variants in samples referred for diagnosis.

### Application

#### Variant discovery.

We sequenced an additional 48 genomic DNA samples from patients with one of multiple monogenic or polygenic phenotypes, making a total of 72 unique samples (supplementary Table IV). Sequencing runs in these samples were performed as in the validation samples described above. In total, we identified 1,929 distinct nonreference sequence variants including 502 exonic coding variants, 645 UTR variants, 6 splicing variants, and 776 intronic and noncoding variants. Approximately 40% of exonic coding variants were synonymous changes (n = 210), whereas the remainder included 275 nonsynonymous single nucleotide variants, 9 frameshift-causing indels, and 8 in-frame indels. Considering all variants, only 48 variants were novel singletons and potentially disease-causing, including 36 nonsynonymous variants, 7 frameshift-causing indels, 3 in-frame indels, and 2 splicing variants. In total, we observed 35,179 sequence changes across these samples, which corresponded to 6,972 exonic coding variants, with 3,536 potentially disease-causing variants. On average, each patient's genome carried 488.1 variants, including 96.8 coding sequence variants, of which 49.1 were potentially deleterious and 2 were novel.

#### Diagnostic yield.

We focused on the ability of our LipidSeq panel to detect genetic variants contributing to patient clinical phenotypes (**Table 4**). From the entire study cohort, 38 patients had no previous sequencing performed: this subgroup comprised our experimental cohort, in which we investigated mutation detection rates based on the disease subgroups existing within the experimental cohort.

**TABLE 4.** Candidate mutation detection rates within experimental dyslipidemia cohorts

	Candidate Mutation	
Disease	Positive	Negative
FH (%) (n = 19)	8 (42.1)	11 (57.9)
HTG (%) (n = 14)	12 (85.7)	2 (14.3)
MODY (%) (n = 2)	1 (50)	1 (50)
FPLD (%) (n = 2)	0 (0)	2 (100)
HBL (%) (n = 1)	1 (100)	0 (0)
Total*^a^* (%) (n = 38)	22 (57.9)	16 (42.1)

a FH patients previously screened for candidate mutations (n = 10) are not included here.

In samples from FH, HTG, MODY, FPLD, and HBL patients, we detected rare nonsynonymous variants (frequency <1%) within 73 candidate genes in 22 patients (57.9%), while 16 patients (42.1%) carried no rare nonsynonymous variants. Subgroup analyses showed that, among 19 patients with suspected FH in whom no previous candidate gene sequencing had been performed, 8 (42.1%) carried heterozygous rare variants in candidate FH genes, including 5 in *LDLR,* 3 in *APOB*, and 1 in *LDLRAP1* (supplementary Table V). Furthermore, 7 of 14 HTG patients (50%) carried rare variants in candidate monogenic HTG genes, which included 1 in *LPL*, 3 in *LMF1*, and 1 each in *GPIHBP1* and *APOA5* (supplementary Table V). Of the HTG patients negative for rare variants in monogenic HTG genes, 5 (35.7%) were carriers of rare variants in polygenic HTG genes, including 4 in *APOB* and 2 in *GCKR* (supplementary Table V). One of two patients referred with suspected MODY carried multiple rare variants in the candidate MODY gene, *CEL* (supplementary Table V). The FPLD patients studied harbored no disease-causing variants in known lipodystrophy genes. Finally, the single HBL patient was heterozygous for a novel nonsense variant in exon 9 of *APOB* (supplementary Table V).

Mutation-negative patients (n = 18) were assessed for the presence of low-frequency variants [minor allele frequency (MAF) < 0.03] in noncandidate dyslipidemia genes included in the LipidSeq panel. A subset of FH patients previously found to be mutation-negative at candidate FH genes was also screened using the LipidSeq panel. Accordingly, several detected rare variants highlighted additional cardio-metabolic pathways with potential relevance to the respective dyslipidemia phenotypes (supplementary Table VI).

## DISCUSSION

In evaluating the performance of our NGS-based dyslipidemia gene resequencing panel, we found that: *1*) combining the Illumina Nextera custom enrichment kit with the MiSeq NGS platform produced high-quality sequencing data resulting in an average (±SD) read depth per base pair of 345.1 (±84.67) with an average >30× coverage for 97.8% of targets; *2*) MiSeq variant detection accuracy was 95.2% in 18 reference samples with known mutations that had been determined using Sanger sequencing; *3*) WGA DNA yielded variant detection rates comparable to Sanger sequencing and thus presents a suitable substitute for NGS when genomic DNA is not available; and *4*) in samples from individuals with a variety of dyslipidemias, including FH, HTG, HBL, MODY, and FPLD, in which no prior sequencing had been performed, 57.9% were identified as carrying at least one candidate variant likely affecting the patient phenotype, which were subsequently confirmed with Sanger sequencing.

Clinical implementation of LipidSeq has several advantages over current Sanger-based clinical resequencing strategies. First, LipidSeq enabled us to process and analyze 12 patient DNA samples within one week, compared with the time requirement several orders of magnitude greater than this to accomplish a comparable amount of screening with Sanger sequencing. On a more limited scale, the stepwise resequencing of only the causative genes for heterozygous FH, namely *LDLR*, *APOB*, and *PCSK9*, can take ∼1 month including bench work and analysis. Similarly, obtaining variant calls from whole exome sequencing can take ∼1 month. Second, we sequenced patient samples for the 23 dyslipidemia genes and 50 other related metabolic genes at a total cost of <$500.00 per sample, which was about half the cost of Sanger sequencing the three candidate FH genes only (∼$1,000.00 per sample). Comparatively, whole exome sequencing on the Illumina HiSeq is approximately three times the cost of the LipidSeq panel (∼$1,700.00 per sample; based on 24 samples per run), with a larger number of patient samples required per run in order to reduce the per-sample cost. Targeted NGS may represent a more economical, focused, and accurate approach for clinical resequencing in monogenic dyslipidemias. These advantages, when considered with the strong concordance in variant detection rates with Sanger sequencing suggest that LipidSeq has the potential to effectively replace Sanger sequencing in the clinic.

The LipidSeq panel also provided supplemental genetic information that could help molecularly diagnose the 42.1% of candidate mutation-negative dyslipidemia patients. Because the LipidSeq panel interrogates genes that act at several points in metabolic pathways associated with dyslipidemias, we detected several low-frequency variants that might play a role in modulating the observed clinical phenotypes. For example, two patients with possible FH carried no variants in FH genes, but instead were carriers of rare variants in the genes encoding *ABCG5* and sortilin 1 (*SORT1*) (supplementary Table VI). Similarly, rare variants in *SORT1* and *ABCG8* were detected in FH patients previously screened for candidate mutations (supplementary Table VI). Genetic variation in *ABCG5* and *ABCG8* has been associated with sitosterolemia while GWAS has recently implicated *SORT1* as a novel locus for LDL cholesterol (24). However, rare variation in *SORT1* has so far not been shown to cause severely elevated LDL cholesterol. While rare variants in noncandidate genes may not currently be of clinical utility, such hypothesis-generating data might be important for future investigations into the polygenic etiology of dyslipidemias, particularly in patients who are mutation-negative in known genes. Furthermore, as the panel is limited to genes involved in lipoprotein metabolism, this targeted approach potentially provides more focus than the unwieldy list of variants from across the genome that would be generated by whole exome sequence analysis of these samples.

In addition to our focus on rare variant detection, we also designed the LipidSeq panel to genotype 178 dyslipidemia-associated SNPs to build polygenic genetic risk scores (GRSs). Targeted sequencing probes were specifically designed because most of the GWAS-derived SNPs lay in intergenic and intronic regions that would not have been genotyped using standard exome sequencing approaches. For potential clinical purposes, we used the top largest effect SNP variants associated with a particular lipid trait identified through the largest GWAS (18). SNPs were grouped based on the strongest associations with lipid traits. For example, the top 14 SNPs previously associated with plasma TG were grouped into a TG GRS. Similar reduced complexity GRSs were determined for HDL cholesterol and LDL cholesterol. These various literature-based GRSs were designed to investigate the polygenic etiology of complex blood lipid traits and cardiovascular endpoints by using SNPs as proxies for assessing the cumulative role of candidate gene variation in estimating cardiovascular risk. Currently, no robust GRSs exist for assessing coronary artery disease risk or plasma lipid concentration; however, a recent study by Talmud et al. (25) successfully applied a 12-SNP LDL GRS in distinguishing mutation-positive FH patients from mutation-negative FH patients. Evaluation of polygenic SNP GRSs concurrent with sequencing data of monogenic dyslipidemia genes may provide additional information that might be useful in some patients. For instance, the GRS may modulate the severity of the phenotype that is primarily determined by a rare variant of large effect in a dyslipidemia gene. Also, there are clearly instances of patients with apparently monogenic forms of dyslipidemia in whom the phenotype is explainable not by a rare mutation of large effect, but rather by a high polygenic GRS, whereby many common variants of individual small effect underlie the phenotype (25). The LipidSeq approach has uniquely integrated GWAS SNP detection with targeted resequencing which provides the potential for more comprehensive risk evaluation that combines both common and rare variation as the next step in evaluating polygenic dyslipidemia.

We also investigated the suitability of WGA DNA samples in the NGS LipidSeq approach, because many samples from interesting patients may be subject to depletion or degradation with time, and require WGA in order to increase DNA mass. We noted objective reductions in WGA DNA sample quality compared with genomic DNA, as indicated by reduced mean coverage (264.5 ± 58.0 vs. 399.4 ± 78.2, *P* = 0.008) and an increased percentage of sequencing targets below minimum 30× coverage (4.0 ± 1.5% vs. 1.0 ± 0.09%, *P* = 0.005). Additionally, the overall concordance between genomic (96%) and WGA DNA (94%) samples as compared with matching Sanger sequencing was significantly different (*P* = 0.008), suggesting a relative quality deficiency with WGA DNA. While the observed differences highlighted a clear distinction between genomic and WGA DNA based on NGS performance metrics, WGA DNA performed at a level approaching the quality of genomic DNA and thus warrants consideration as an alternative DNA source when high molecular weight genomic DNA is not available.

The advantages of LipidSeq arise largely from the hypothesis-driven approach to genomic screening, which interrogates genetic variation at known dyslipidemia loci. Also, to date the yield of new genes in lipoprotein metabolism discovered by whole exome sequencing of large numbers of dyslipidemic individuals has been somewhat underwhelming, further suggesting that the whole exome approach may not be required for routine clinical evaluation of most patients with monogenic dyslipidemias (26–28). By focusing the lens of variant detection upon established loci, the process of developing a molecular diagnosis becomes less convoluted in comparison to an agnostic whole exome sequencing approach. Although potentially deleterious mutations that have no clear effect on patient phenotypes are likely to be detected, the scale of variants of unknown significance detected is clearly much reduced using a targeted approach rather than the whole exome sequencing approach. While it might be possible to target genes in metabolic pathways using bioinformatic masking of whole exome sequence results, the LipidSeq approach still has the advantages of lower cost and faster turnover time. Finally, LipidSeq was developed using Illumina NGS protocols. Recently, the US Food and Drug Administration approved marketing authorization for the Illumina MiSeqDx platform (7). This endorsement supports the quality and reliability of Illumina NGS technology and improves the outlook for standardized clinical NGS for dyslipidemias.

Implementation of the LipidSeq panel revealed some limitations that are common across NGS approaches to screening for disease-causing mutations. First, we consistently observed low coverage (<30×) or failed target coverage at the UTR and other noncoding regions, as well as regions rich in guanine-cytosine base content. These regions were often the sites of discordant variant calling during our validation stage analysis. However, these regions are largely noncoding and are therefore of somewhat less interest when searching for possible disease-causing mutations (4). Second, NGS-based approaches do not robustly detect structural and copy number variation, because inversions and translocations tend to occur near repetitive sequences (29). Our current analytical pipeline utilizes a structural variation tool within the CLC Bio Genomics Workbench; however, further assessment of the performance of this tool is required before routine implementation in clinical resequencing. Finally, ethical issues have emerged regarding incidental genomic findings and the extent to which mutation detection results are disclosed to the patient, particularly when potentially disease-causing and clinically actionable mutations are detected but may be unrelated to the immediate patient health issue (30–32). In response to this issue, the American College of Medical Genetics and Genomics (ACMG) identified 56 genes associated with adult-onset diseases for which incidental genomic findings should be disclosed to patients (33). As the only three ACMG genes featured on the LipidSeq panel include the known FH genes *LDLR, APOB*, and *PCSK9*, the LipidSeq approach considerably limits patient exposure to incidental genomic findings unrelated to dyslipidemia.

In summary, we report the first comprehensive targeted NGS approach for molecular diagnoses across the spectrum of monogenic dyslipidemias. The panel performs well, with high concordance in samples with known mutations based on Sanger sequencing and a high detection rate of mutations likely to be causative for disease in samples not previously sequenced. Clinical implementation of LipidSeq has the potential to diagnose patients with monogenic dyslipidemias with a high degree of speed and accuracy and at lower cost than either Sanger sequencing or whole exome sequencing. Furthermore, targeted NGS of dyslipidemia-related loci will help to provide a more focused picture of monogenic and polygenic contributors that underlie dyslipidemia, and will not provide unwanted information about incidental pathogenic, clinically actionable variants in nonmetabolic pathways that affect disease risk. A significant limitation continues to center on the interpretation and parsing of detected variants based on clinical utility; however, as comprehensive genomic variation in dyslipidemia patients continues to be documented, we stand to gain greater insight into the spectrum of variants underlying the phenotypic heterogeneity commonly observed within dyslipidemia subtypes.

## Supplementary Material

Supplemental Data
