# Supplementary material for: LipidSeq: a next-generation clinical resequencing panel for monogenic dyslipidemias
Source: J Lipid Res. 2014 Apr;55(4):765–72. doi: 10.1194/jlr.D045963 (PMC3966710; doi:10.1194/jlr.D045963)
Supplement: Supplemental Data [file supp_55_4_765__index.html]

LipidSeq: a next-generation clinical resequencing panel for monogenic dyslipidemias — LipidSeq: a next-generation clinical resequencing panel for monogenic dyslipidemias — Supplemental Data 

# LipidSeq: a next-generation clinical resequencing panel for monogenic dyslipidemias

## Supplemental Data

**Files in this Data Supplement:**

- Supplemental Tables - Supplemental tables
