# Supplementary material for: LipidSeq: a next-generation clinical resequencing panel for monogenic dyslipidemias
Source: J Lipid Res. 2014 Apr;55(4):765–72. doi: 10.1194/jlr.D045963 (PMC3966710; doi:10.1194/jlr.D045963)
Supplement: Supplemental Data [file supp_D045963_jlr.D045963-1.pdf]

SUPPLEMENTAL TABLES

Supplemental Table 1. Genes sequenced using the custom enrichment panel.

Gene	Chr	Start[a]	Stop[a]	RefSeq	Size (bp)	Phenotype
**Established monogenic dyslipidemia genes**						
LDLRAP1	1	25870071	25895377	NM_015627	8,188	FH
PCSK9	1	55505149	55530526	NM_174936	10,607	FH
ANGPTL3	1	63063158	63071976	NM_014495	6,726	FC hypolipidemia
APOB	2	21224301	21266945	NM_000384	24,273	FH, HHBL
ABCG5	2	44039611	44066039	NM_022436	8,153	Sitosterolemia
ABCG8	2	44066103	44105947	NM_022437	8,001	Sitosterolemia
MTTP	4	100485240	100545154	NM_000253	12,749	ABL
SAR1B	5	133936839	133968533	NM_001033503	11,532	Anderson disease
LPL	8	19796582	19824770	NM_000237	9,247	HTG
GPIHBP1	8	144295068	144299044	NM_178172	5,029	HTG
ABCA1	9	107543283	107690527	NM_005502	27,554	Low HDL-C
LIPA	10	90973326	91011660	NM_001127605	8,157	CESD; Wolman syndrome
APOA5	11	116660086	116663136	NM_052968	5,323	HTG
APOC3	11	116700624	116703787	NM_000040	3,735	HTG
APOA1	11	116706467	116708338	NM_000039	4,381	Low HDL-C
GPD1	12	50497602	50505103	NM_005276	7,699	HTG
SCARB1	12	125262174	125348519	NM_005505	9,786	High HDL-C
LIPC	15	58702953	58861073	NM_000236	7,089	Hepatic lipase deficiency
LMF1	16	903634	1031318	NM_022773	10,794	HTG
CETP	16	56995835	57017757	NM_000078	8,537	High HDL-C
LCAT	16	67973787	67978656	NM_000229	3,445	Low HDL-C
LDLR	19	11200037	11244506	NM_000527	12,804	FH
APOE	19	45409039	45412650	NM_000041	4,511	HTG
APOC2	19	45449239	45452822	NM_000483	4,224	HTG
**Other candidate dyslipidemia genes**						
**Gene**	**Chr**	**Start[a]**	**Stop[a]**	**RefSeq**	**Size (bp)**	**Phenotype**
SORT1	1	109852187	109940563	NM_002959	17,839	GWAS LDL-C candidate gene
GALNT2	1	230202956	230417875	NM_004481	11,762	GWAS TG candidate gene
GCKR	2	27719470	27746556	NM_001486	9,717	GWAS TG candidate gene
MYLIP	6	16129277	16148479	NM_013262	7,621	GWAS LDL-C candidate gene
NPC1L1	7	44552134	44580914	NM_013389	12,925	Ezetimibe target
MLXIPL	7	73007524	73038903	NM_032951	9,590	GWAS TG candidate
TRIB1	8	126442563	126450647	NM_025195	7,341	GWAS TG candidate
PNPLA2	11	818901	825573	NM_020376	6,733	Neutral Lipid Storage Disease
APOA4	11	116691418	116694011	NM_000482	3,360	TG candidate
LIPG	18	47087069	47119278	NM_006033	9,833	High HDL-C
CREB3L3	19	4153598	4173051	NM_032607	7,867	HTG-associated gene
LIPE	19	42905664	42931578	NM_005357	9,112	TG candidate gene
PLTP	20	44527259	44541003	NM_006227	7,677	HDL-C candidate gene
ABCG1	21	43619799	43724497	NM_004915	17,085	Low HDL-C candidate gene

[a]Start and stop positions were obtained from the University of California Santa Cruz (UCSC) genome browser using the February 2009 GRCh37/hg19 genome build. abbreviations: CHR, chromosome; FC, familial combined, FH, familial hypercholesterolemia; GWAS, genome-wide association study; HDL, high-density lipoprotein; LDL, low-density lipoprotein; MODY, maturity onset diabetes of the young; HHBL, homozygous hypobetalipoproteinemia; HTG, hypertriglyceridemia; ABL, abetalipoproteinemia; CESD, cholesterol ester storage disease; MD, muscular dystrophy

Supplemental Table 1 continued. Genes sequenced using the custom enrichment panel.

Miscellaneous dyslipidemia genes						
**Gene**	**Chr**	**Start[a]**	**Stop[a]**	**RefSeq**	**Size (bp)**	**Phenotype**
ZMPSTE24	1	40723722	40759856	NM_005857	8,654	Lipodystrophy and progeria
CPT2	1	53662101	53679869	NM_000098	6,994	Statin-induced myopathy
AMPD1	1	115215719	115238239	NM_000036	9,438	Statin-induced myopathy
LMNA	1	156052369	156109880	NM_170707	14,450	Partial lipodystrophy
KLF11	2	10170776	10194963	NM_003597	9,848	MODY subtype 7
LPIN1	2	11817705	11967535	NM_001261428	18,824	Lipodystrophy candidate
NEUROD1	2	182540833	182545392	NM_002500	5,566	MODY subtype 6
CIDEC	3	9908394	9921938	NM_001199623	6,413	Partial lipodystrophy
PPARG	3	12329349	12475855	NM_015869	10,057	Partial lipodystrophy
COQ2	4	84184972	84206067	NM_015697	6,641	Statin-induced myopathy
GCK	7	44183870	44229022	NM_033508	10,472	MODY subtype 2
CAV2	7	116139655	116148595	NM_001233	6,418	Other
CAV1	7	116164839	116201239	NM_001753	7,245	Generalized lipodystrophy
PAX4	7	127250346	127255982	NM_006193	7,308	MODY subtype 9
BLK	8	11351521	11422108	NM_001715	11,279	MODY subtype 11
WRN	8	30890778	31031277	NM_000553	18,427	Werner syndrome
CEL	9	135936741	135947250	NM_001807	7,762	Inherited diabetes
AGPAT2	9	139567595	139581911	NM_006412	5,815	Generalized lipodystrophy
INS	11	2181009	2182439	NM_001185097	3,244	MODY subtype 10
KCNJ11	11	17406796	17410878	NM_000525	6,211	Transient neonatal diabetes
ABCC8	11	17414432	17498449	NM_000352	18,450	Transient neonatal diabetes
BSCL2	11	62457734	62477091	NM_001122955	7,247	Generalized lipodystrophy
SLC22A8	11	62760296	62783317	NM_004254	7,105	Statin-induced myopathy
PYGM	11	64513861	64528187	NM_005609	10,850	Statin-induced myopathy
HNF1A	12	121415861	121440315	NM_000545	9,100	MODY subtype 3
PDX1	13	28494168	28500451	NM_000209	5,423	MODY subtype 4
PLIN1	15	90207598	90222648	NM_002666	7,715	Partial lipodystrophy
HNF1B	17	36046434	36105096	NM_000458	8,141	Type 2 diabetes
PTRF	17	40554467	40575506	NM_012232	6,734	Generalized lipodystrophy
LMNB2	19	2428163	2456966	NM_032737	9,933	Partial lipodystrophy
AKT2	19	40736224	40791443	NM_001626	13,013	Partial lipodystrophy
HNF4A	20	42984441	43061485	NM_000457	13,787	MODY subtype 1
CYP2D6	22	42522501	42526883	NM_000106	6,242	Statin-induced myopathy
PPARA	22	46546458	46639653	NM_005036	15,333	Statin-induced myopathy
EMD	X	153607597	153609883	NM_000117	5,002	Emery-Dreifuss MD

[a]*Start and stop positions were obtained from the University of California Santa Cruz (UCSC) genome browser using the February 2009 GRCh37/hg19 genome build. abbreviations: Chr, chromosome; FC, familial combined, FH, familial hypercholesterolemia; GWAS, genome-wide association study; HDL, high-density lipoprotein; LDL, low-density lipoprotein; MODY, maturity onset diabetes of the young; HHBL, homozygous hypobetalipoproteinemia; HTG, hypertriglyceridemia; ABL, abetalipoproteinemia; CESD, cholesterol ester storage disease; MD, muscular dystrophy*

Supplemental Table 2. SNPs genotyped using the custom enrichment panel.

Locus	rsID	Chr	Position
LDLRAP1	rs12027135	1	25775733
PABPC4	rs4660293	1	40028180
PCSK9	rs11206510	1	55496039
PCSK9	rs2479409	1	55504650
ANGPTL3	rs2131925	1	63025942
ANGPTL3	rs3850634	1	63050598
ANGPTL3	rs10889353	1	63118196
EVI5	rs7515577	1	93009438
CELSR2, PSRC1, SORT1	rs12740374	1	109817590
SORT1	rs629301	1	109818306
Factor V	rs6025	1	169519049
ZNF648	rs1689800	1	182168885
MOSC1	rs2807834	1	220970593
MOSC1	rs2642442	1	220973563
MIA3	rs17465637	1	222823529
GALNT2	rs4846914	1	230295691
GALNT2	rs1321257	1	230305312
IRF2BP2	rs514230	1	234858597
APOB	rs7557067	2	21208211
APOB	rs1042034	2	21225281
APOB	rs1367117	2	21263900
APOB	rs515135	2	21286057
GCKR	rs1260326	2	27730940
ABCG5/8	rs4299376	2	44072576
ABCG8	rs6544713	2	44073881
RAB3GAP1	rs7570971	2	135837906
RAB3GAP1	rs6759321	2	136322676
COBLL1	rs10195252	2	165513091
COBLL1	rs12328675	2	165540800
IRS1	rs2943645	2	227099180
IRS1	rs2972146	2	227100698
IRS1	rs1515100	2	227128917
PPARG	rs1801282	3	12393125
RAF1	rs2290159	3	12628920
MSL2L1	rs645040	3	135926622
KLHL8	rs442177	4	88030261
SLC39A8	rs13107325	4	103188709
ARL15	rs6450176	5	53298025
MAP3K1	rs9686661	5	55861786
HMGCR	rs3846663	5	74655726
HMGCR	rs12916	5	74656539

Supplemental Table 2 continued. SNPs genotyped using the custom enrichment panel.

Locus	rsID	Chr	Position
TIMD4	rs6882076	5	156390297
TIMD4-HAVCR1	rs1501908	5	156398169
TIMD4	rs1553318	5	156479323
MYLIP	rs3757354	6	16127407
HFE	rs1800562	6	26093141
HLA	rs2247056	6	31265490
HLA	rs3177928	6	32412435
C6orf106	rs2814982	6	34546560
C6orf106	rs2814944	6	34552797
FRK	rs9488822	6	116312893
FRK	rs11153594	6	116354591
CITED2	rs605066	6	139829666
LPA	rs1564348	6	160578860
LPA	rs10455872	6	161010118
LPA	rs1084651	6	161089817
DNAH11	rs2285942	7	21582917
DNAH11	rs12670798	7	21607352
NPC1L1	rs2072183	7	44579180
NPC1L1	rs217386	7	44600695
TYW1B	rs13238203	7	72129667
MLXIPL	rs714052	7	72864869
MLXIPL	rs7811265	7	72934510
MLXIPL	rs17145738	7	72982874
KLF14	rs4731702	7	130433384
PPP1R3B	rs9987289	8	9183358
PPP1R3B	rs2126259	8	9185146
PINX1	rs11776767	8	10683929
XKR6-AMAC1L2	rs7819412	8	11045161
NAT2	rs1961456	8	18255709
NAT2	rs1495741	8	18272881
LPL S447X	rs328	8	19819724
LPL	rs12678919	8	19844222
CYP7A1	rs1030431	8	59311697
CYP7A1	rs2081687	8	59388565
TRPS1	rs2293889	8	116599199
TRPS1	rs2737229	8	116648565
TRIB1	rs2954022	8	126482621
TRIB1	rs2954029	8	126490972
TRIB1	rs10808546	8	126495818
PLEC1	rs11136341	8	145043543
TTC39B	rs471364	9	15289578

Supplemental Table 2 continued. SNPs genotyped using the custom enrichment panel.

Locus	rsID	Chr	Position
TTC39B	rs643531	9	15296034
TTC39B	rs581080	9	15305378
ANRIL	rs10757274	9	22096055
CDKN2A/2B	rs10811661	9	22134094
ABCA1	rs1883025	9	107664301
ABO	rs651007	9	136153875
ABO	rs649129	9	136154304
ABO	rs635634	9	136155000
CXCL12	rs501120	10	44753867
JMJD1C	rs10761731	10	65027610
CYP26A1	rs2068888	10	94839642
GPAM	rs1129555	10	113910721
GPAM	rs2255141	10	113933886
TCF7L2	rs7903146	10	114758349
AMPD3	rs2923084	11	10388782
SPTY2D1	rs10128711	11	18632984
SPTY2D1	rs10832963	11	18664241
LRP4	rs3136441	11	46743247
FADS1–2–3	rs174546	11	61569830
FADS1–2–3	rs174547	11	61570783
FADS1–2–3	rs174550	11	61571478
FADS1–2–3	rs174583	11	61609750
FADS1–2–3	rs174601	11	61623140
APOA1-C3-A4-A5	rs964184	11	116648917
APOA1-C3-A4-A5	rs3135506	11	116662407
APOA1-C3-A4-A5	rs662799	11	116663707
UBASH3B	rs7941030	11	122522375
UBASH3B	rs7115089	11	122530591
ST3GAL4	rs11220462	11	126243952
ST3GAL4	rs11220463	11	126248211
PDE3A	rs7134375	12	20473758
SLCO1B1	rs4149056	12	21331549
SLCO1B1	rs4363657	12	21368722
LRP1	rs11613352	12	57792580
LRP1	rs3741414	12	57844049
MMAB, MVK	rs2338104	12	109895168
MVK	rs7134594	12	110000193
BRAP	rs11065987	12	112072424
HNF1A	rs2650000	12	121388962
HNF1A	rs1169288	12	121416650
SBNO1	rs4759375	12	123796238
ZNF664	rs4765127	12	124460167

Supplemental Table 2 continued. SNPs genotyped using the custom enrichment panel.

Locus	rsID	Chr	Position
ZNF664	rs12310367	12	124486678
SCARB1	rs838880	12	125261593
NYNRIN	rs2332328	14	24883058
NYNRIN	rs8017377	14	24883887
CAPN3	rs2412710	15	42683787
FRMD5	rs2929282	15	44245931
LIPC	rs10468017	15	58678512
LIPC	rs1532085	15	58683366
LIPC	rs261342	15	58731153
LACTB	rs2652834	15	63396867
CTF1	rs11649653	16	30918487
FTO	rs1421085	16	53800954
CETP	rs173539	16	56988044
CETP	rs247616	16	56989590
CETP	rs3764261	16	56993324
CETP	rs7205804	16	57004889
LCAT	rs2271293	16	67902070
LCAT	rs16942887	16	67928042
HPR	rs2000999	16	72108093
CMIP	rs2925979	16	81534790
STARD3	rs881844	17	37810218
STARD3	rs11869286	17	37813856
OSBPL7	rs7225700	17	45391804
OSBPL7	rs7206971	17	45425115
ABCA8	rs4148008	17	66875294
PGS1	rs4082919	17	76377482
PGS1	rs4129767	17	76403984
LIPG	rs7241918	18	47160953
LIPG	rs7239867	18	47164717
LIPG	rs4939883	18	47167214
MC4R	rs12967135	18	57849023
ANGPTL4	rs7255436	19	8433196
ANGPTL4	rs2967605	19	8469738
LDLR	rs6511720	19	11202306
LOC55908	rs737337	19	11347493
CILP2	rs10401969	19	19407718
NCAN, CILP2, PBX4	rs17216525	19	19662220
APOE–C1–C2	rs439401	19	45414451
APOE–C1–C2	rs4420638	19	45422946
FLJ36070	rs492602	19	49206417
LILRA3	rs386000	19	54792761

Supplemental Table 2 continued. SNPs genotyped using the custom enrichment panel.

Locus	rsID	Chr	Position
ERGIC3	rs2277862	20	34152782
MAFB	rs2902940	20	39091487
MAFB	rs2902941	20	39091514
MAFB	rs6102059	20	39228784
TOP1	rs6029526	20	39672618
TOP1	rs4297946	20	39811275
TOP1	rs909802	20	39936815
HNF4A	rs1800961	20	43042364
PLTP	rs4810479	20	44545048
PLTP	rs6065906	20	44554015
PLTP	rs7679	20	44576502
UBE2L3	rs181362	22	21932068
PLA2G6	rs5756931	22	38546033

Supplemental Table 3. Breakdown of discordant calls between technical replicates.

		Variants identified in both original and replicate		Variants identified in either original or replicate	
**Sample ID**	**Input DNA**	**Concordant**	**Discordant**	**Original**	**Replicate**
3344	genomic	469	3	7	6
	WGA	460	5	14	9
3645	genomic	471	4	10	6
	WGA	461	3	21	6
3732	genomic	501	2	10	6
	WGA	482	4	27	6
4221	genomic	477	2	5	8
	WGA	473	2	9	7
4270	genomic	426	3	4	10
	WGA	417	4	12	9
4667	genomic	443	5	5	12
	WGA	427	3	23	8

Abbreviations: WGA, whole genome amplified.

Supplemental Table 4. Breakdown of variants identified in unique study samples.

			Variant type					Coding variant effect				
Sample ID	Phenotype	Category	Coding	Splice	UTR	Noncoding	Total	Nonsyn	Syn	Frameshift	Inframe	Total
4667	HTG	Validation	94	0	129	230	453	43	51	0	0	94
4270	HTG	Validation	83	1	126	224	434	41	40	0	2	83
4221	HTG	Validation	98	0	138	249	485	46	51	0	1	98
4047	HTG	Validation	98	0	132	251	481	49	47	0	2	98
3965	HTG	Validation	100	1	152	220	473	49	49	1	1	100
3891	HTG	Validation	101	1	158	235	495	50	50	0	1	101
3760	HTG	Validation	90	1	121	252	464	47	42	0	1	90
3746	HTG	Validation	97	1	138	229	465	49	47	0	1	97
3732	HTG	Validation	96	1	170	247	514	46	49	0	1	96
3645	HTG	Validation	100	0	145	240	485	51	47	0	2	100
3609	HTG	Validation	94	0	147	262	503	50	43	1	0	94
3345	HTG	Validation	89	1	153	236	479	43	45	0	1	89
3344	HTG	Validation	90	0	142	245	477	45	43	0	2	90
3141	HTG	Validation	100	0	151	240	491	50	48	0	2	100
4063	HTG	Validation	98	1	131	258	488	53	44	0	1	98
4135	HTG	Validation	95	0	166	247	508	48	46	0	1	95
3751	HTG	Validation	101	1	120	258	480	53	47	0	1	101
2769	HTG	Validation	99	1	158	243	501	47	50	0	2	99
11588	FPLD	Validation	100	1	124	259	484	55	43	0	2	100
9040	Low HDL	Validation	101	0	158	256	515	49	50	0	2	101
9043	FH	Validation	91	0	169	271	531	43	48	0	0	91
6399	FH	Validation	96	1	140	242	479	50	44	0	2	96
9025	ABL	Validation	97	0	123	227	447	45	49	0	3	97
8926	ABL	Validation	102	2	137	249	490	52	49	0	1	102
4631	FH	Monogenic	102	1	186	246	535	53	47	0	2	102
10247	FH	Monogenic	97	1	143	225	466	49	45	1	2	97
10248	FH	Monogenic	96	0	154	230	480	49	46	1	0	96
10249	FH	Monogenic	85	0	118	222	425	43	41	0	1	85
10250	FH	Monogenic	93	1	139	255	488	49	41	1	2	93
11538	FH	Monogenic	95	0	125	236	456	44	50	0	1	95
11547	FH	Monogenic	107	0	157	234	498	54	51	0	2	107
11619	FH	Monogenic	98	1	142	275	516	43	54	0	1	98
11634	FH	Monogenic	88	1	151	243	483	39	48	0	1	88
11636	FH	Monogenic	96	0	189	259	544	48	46	0	2	96
11659	FH	Monogenic	85	0	134	235	454	44	40	0	1	85

Abbreviations as in Supplemental Table 1, plus UTR, untranslated region; Nonsyn, nonsynonymous variant; Syn, synonymous variant.

Supplemental Table 4 continued. Breakdown of variants identified in unique study samples.

			Variant type					Coding variant effect				
**Sample ID**	**Phenotype**	**Category**	**Coding**	**Splice**	**UTR**	**Noncoding**	**Total**	**Nonsyn**	**Syn**	**Frameshift**	**Inframe**	**Total**
11660	FH	Monogenic	107	1	131	240	479	51	54	0	2	107
11718	FH	Monogenic	93	0	135	263	491	48	44	0	1	93
11783	FH	Monogenic	106	1	160	262	529	48	55	0	3	106
11823	FH	Monogenic	91	0	193	231	515	47	43	0	1	91
11824	FH	Monogenic	107	0	145	255	507	52	55	0	0	107
11825	FH	Monogenic	87	1	120	237	445	47	38	0	2	87
8852	FH	Monogenic	103	1	135	243	482	48	54	0	1	103
9075	FH	Monogenic	96	0	156	238	490	48	47	0	1	96
10018	FH_old[a]	Monogenic	94	1	133	245	473	44	48	0	2	94
9885	FH_old	Monogenic	74	0	136	236	446	36	37	0	1	74
9035	FH_old	Monogenic	112	1	166	245	524	58	53	0	1	112
8836	FH_old	Monogenic	92	1	146	235	474	42	48	0	2	92
8566	FH_old	Monogenic	112	1	123	240	476	52	58	1	1	112
8534	FH_old	Monogenic	97	1	130	237	465	49	47	0	1	97
6455	FH_old	Monogenic	110	1	129	253	493	57	51	0	2	110
4621	FH_old	Monogenic	91	0	138	224	453	46	44	0	1	91
4205	FH_old	Monogenic	90	0	153	249	492	40	49	0	1	90
3457	FH_old	Monogenic	98	0	139	242	479	46	50	0	2	98
11845	HBL	Monogenic	98	0	188	243	529	42	54	0	2	98
11618	FPLD	Monogenic	87	0	124	230	441	45	42	0	0	87
11816	FPLD	Monogenic	94	0	168	240	502	48	45	0	1	94
11848	HTG	Polygenic	89	0	149	214	452	37	50	0	2	89
11849	HTG	Polygenic	89	0	121	228	438	42	45	1	1	89
11565	HTG	Polygenic	93	1	160	246	500	46	45	0	2	93
11620	HTG	Polygenic	92	0	144	251	487	45	46	0	1	92
11635	HTG	Polygenic	101	0	151	234	486	46	54	0	1	101
11637	HTG	Polygenic	97	0	132	258	487	44	51	0	2	97
11696	HTG	Polygenic	111	0	138	263	512	55	53	1	2	111
11705	HTG	Polygenic	113	0	128	258	499	53	58	1	1	113
11585	HTG	Polygenic	103	0	147	268	518	53	49	0	1	103
11586	HTG	Polygenic	95	0	145	253	493	46	47	0	2	95
11601	HTG	Polygenic	89	0	147	263	499	45	44	0	0	89
11617	HTG	Polygenic	111	1	180	284	576	63	47	0	1	111
11834	HTG	Polygenic	107	0	164	276	547	48	58	0	1	107

[a] *"FH_old" refers to patient samples with previous sequencing in FH candidate genes. Abbreviations as in Table Supplemental Table 1.*

Supplemental Table 4 continued. Breakdown of variants identified in unique study samples.

			Variant type					Coding variant effect				
**Sample ID**	**Phenotype**	**Category**	**Coding**	**Splice**	**UTR**	**Noncoding**	**Total**	**Nonsyn**	**Syn**	**Frameshift**	**Inframe**	**Total**
11837	HTG	Polygenic	93	0	155	258	506	43	49	0	1	93
3217	MODY	Polygenic	113	1	146	237	497	62	50	1	0	113
5725	MODY	Polygenic	85	1	156	250	492	42	43	0	0	85
		Totals:	6972	33	10477	17659	35141	3433	3436	10	93	6972

Abbreviations as in Supplemental Table 1.

Supplemental Table 5. Candidate gene variants detected in suspected monogenic dyslipidemia patients

Class	Gene	Exon	rsID	Variant position		Variant frequency		*in silico* prediction		HGMD
				Nucleotide	Amino acid	ESP	1KG	SIFT	PolyPhen2	
FH	*APOB*	3	NA	c.C218A	p.A73D	0.000077	NA	Tolerated	Benign	No
		26	rs148498577	c.T8550G	p.I2850M	0.000461	NA	Tolerated	Benign	No
		26	rs72653087	c.6639_6641del	p.2213_2214del	NA	NA	NA	NA	No
	LDLR	3	rs144172724	c.G301A	p.E101K	0.000077	NA	Damaging	Probably damaging	Yes
		6	NA	c.820delA	p.T274fs	NA	NA	NA	NA	Yes
		10	rs139617694	c.1359-1G>A	NA	NA	NA	NA	NA	Yes
		12	rs137929307	c.G1775A	p.G592E	0.000231	NA	Damaging	Probably damaging	Yes
		17	NA	c.G2479A	p.V827I	0.000769	NA	Tolerated	Probably damaging	Yes
	LDLRAP1	3	NA	c.G262A	p.V88M	NA	NA	Damaging	Probably damaging	No
HTG	*ANGPTL3*	4	rs77871363	c.T776C	p.M259T	0.016303	NA	Tolerated	Benign	No
	APOA5	4	NA	c.G655C	p.A219P	NA	NA	Tolerated	Possibly damaging	No
	APOB	26	rs533617	c.A5768G	p.H1923R	0.027603	0.05	Damaging	Possibly damaging	Yes
		26	NA	c.A9838T	p.M3280L	NA	NA	Damaging	Probably damaging	No
		26	rs1042023	c.C10294G	p.Q3432E	0.008688	0.01	Tolerated	Possibly damaging	Yes
		26	rs61744153	c.C11477T	p.T3826M	0.001922	0.0026	Damaging	Possibly damaging	No
		29	rs72654423	c.A12940G	p.I4314V	0.006844	0.01	Tolerated	Benign	No
	GCKR	13	NA	c.1134_1135insA	p.L378fs	NA	NA	NA	NA	No
		18	rs8179249	c.G1619A	p.R540Q	0.005305	0.01	Tolerated	Benign	No
	GPIHBP1	1	NA	c.G13A	p.G5R	NA	NA	Tolerated	Benign	No
	LMF1	2	NA	c.G196T	p.V66L	NA	NA	Tolerated	NA	No

Supplemental Table 5 continued. Candidate gene variants detected in suspected monogenic dyslipidemia patients

				Variant position		Variant frequency		*in silico* prediction		
**Class**	**Gene**	**Exon**	**rsID**	**Nucleotide**	**Amino acid**	**ESP**	**1KG**	**SIFT**	**PolyPhen2**	**HGMD**
HTG	*LMF1*	7	rs143076454	c.C1060T	p.R354W	0.009556	0.03	Tolerated	NA	No
		8	rs35168378	c.G1091A	p.R364Q	0.028904	0.03	Tolerated	NA	No
		11	rs4984948	c.C1685G	p.P562R	0.008152	0.01	Tolerated	NA	No
	LPL	2	rs1801177	c.G106A	p.D36N	0.027064	0.01	NA	Possibly damaging	Yes
		5	NA	c.G644A	p.G215E	0.000384	NA	NA	Possibly damaging	Yes
MODY	*CEL*	10	rs77696629	c.T1463C	p.I488T	NA	NA	Damaging	Possibly damaging	No
		11	NA	c.2039_2040insC	p.G680fs	NA	NA	NA	NA	No
		11	NA	c.A2119C	p.T707P	0.002129	NA	Tolerated	Possibly damaging	No
		11	NA	c.A2138G	p.E713G	NA	NA	Tolerated	Possibly damaging	No
		11	NA	c.A2140G	p.T714A	NA	NA	Tolerated	Benign	No
		11	NA	c.G2143C	p.A715P	NA	NA	Tolerated	Possibly damaging	No
		11	rs201411101	c.C2152A	p.P718T	NA	NA	Damaging	Possibly damaging	No
HBL	*APOB*	9	NA	c.C1018T	p.Q340X	NA	NA	Tolerated	Possibly damaging	No

Abbreviations as in Supplemental Tables 1-4; plus ESP, Exome Sequencing Project; 1KG, 1000 Genomes database; SIFT, Sorting Intolerant From Tolerant software; HGMD, Human Genetic Mutation Database.

Supplemental Table 6. Genetic variants identified in candidate gene mutation-free dyslipidemia patients.

				Variant position		Variant frequency		*in silico* prediction		
**Class**	**Gene**	**Exon**	**rsID**	**Nucleotide**	**Amino acid**	**ESP**	**G1K**	**SIFT**	**PolyPhen2**	**HGMD**
FH	*ABCA1*	7	rs141420090	c.T551C	p.L184S	0.000077	NA	Tolerated	Benign	No
FH	*ABCA1*	11	rs9282543	c.T1196C	p.V399A	0.003844	0.004	Tolerated	Possibly damaging	Yes
FH	*ABCA1*	12	rs148314522	c.C1338G	p.D446E	0.000154	NA	Tolerated	Benign	No
FH	*ABCA1*	24	rs33918808	c.G3516C	p.E1172D	0.075581	0.03	Tolerated	Benign	Yes
FH	*ABCC8*	36	NA	c.G4326C	p.E1442D	NA	NA	Damaging	Probably damaging	No
FH	*ABCG5*	1	rs56204478	c.G80C	p.G27A	0.00377	NA	Tolerated	Benign	No
FH	*ABCG8*	4	NA	c.C450A	p.H150Q	NA	NA	Damaging	Probably damaging	No
FH	*AMPD1*	7	rs34526199	c.A959T	p.K320I	0.024143	0.05	Damaging	Probably damaging	Yes
FH	*AMPD1*	8	rs61752478	c.G1029T	p.M343I	0.003844	0.0026	Damaging	Probably damaging	Yes
FH	*AMPD1*	15	NA	c.T2135C	p.M712T	NA	NA	Damaging	Possibly damaging	No
FH	*APOA4*	3	rs12721043	c.G481T	p.A161S	0.007851	0.01	Tolerated	Possibly damaging	Yes
FH	*APOB*	15	rs12691202	c.G2188A	p.V730I	0.02545	0.05	Tolerated	Benign	Yes
FH	*APOB*	22	rs12713844	c.G3337C	p.D1113H	0.00815	0.01	Damaging	Possibly damaging	Yes
FH	*APOB*	26	rs1801699	c.A5741G	p.N1914S	0.01338	0.02	Damaging	Possibly damaging	Yes

Abbreviations as in Supplemental Table 1; and ESP, Exome Sequencing Project; 1KG, 1000 Genomes database; SIFT, Sorting Intolerant From Tolerant software; HGMD, Human Genetic Mutation Database.

Supplemental Table 6 continued. Genetic variants identified in candidate gene mutation-free dyslipidemia patients

				Variant position		Variant frequency		*in silico* prediction		
**Class**	**Gene**	**Exon**	**rsID**	**Nucleotide**	**Amino acid**	**ESP**	**G1K**	**SIFT**	**PolyPhen2**	**HGMD**
FH	*APOB*	29	rs1801695	c.G13441A	p.A4481T	0.028525	0.03	Tolerated	Benign	Yes
FH	*APOB*	29	rs1801702	c.G12809C	p.R4270T	0.04921	0.03	Tolerated	Possibly damaging	No
FH	*BSCL2*	7	rs147902831	c.A683G	p.E228G	NA	NA	Tolerated	Probably damaging	No
FH	*CEL*	10	rs77696629	c.T1463C	p.I488T	NA	NA	Damaging	Possibly damaging	No
FH	*CEL*	11	NA	c.A2138G	p.E713G	NA	NA	Tolerated	Possibly damaging	No
FH	*CEL*	11	NA	c.A2140G	p.T714A	NA	NA	Tolerated	Benign	No
FH	*CEL*	11	NA	c.G2143C	p.A715P	NA	NA	Tolerated	Possibly damaging	No
FH	*CEL*	11	rs201411101	c.C2152A	p.P718T	NA	NA	Damaging	Possibly damaging	No
FH	*CEL*	11	NA	c.A2119C	p.T707P	0.00213	NA	Tolerated	Possibly damaging	No
FH	*CEL*	11	NA	c.A2138G	p.E713G	NA	NA	Tolerated	Possibly damaging	No
FH	*CEL*	11	NA	c.A2140G	p.T714A	NA	NA	Tolerated	Benign	No
FH	*CEL*	11	NA	c.G2143C	p.A715P	NA	NA	Tolerated	Possibly damaging	No
FH	*CEL*	11	rs201411101	c.C2152A	p.P718T	NA	NA	Damaging	Possibly damaging	No
FH	*CETP*	15	rs1800777	c.G1403A	p.R468Q	0.028547	0.04	Tolerated	Benign	Yes
FH	*COQ2*	1	rs112033303	c.A64T	p.R22X	0.016797	0.02	Tolerated	Probably damaging	No

Supplemental Table 6 continued. Genetic variants identified in candidate gene mutation-free dyslipidemia patients

Class	Gene	Exon	rsID	Variant position		Variant frequency		*in silico* prediction		HGMD
				Nucleotide	Amino acid	ESP	G1K	SIFT	PolyPhen2	
FH	*CPT2*	4	rs144760921	c.G500A	p.R167Q	0.000231	NA	Tolerated	Possibly damaging	No
FH	*CPT2*	4	rs144658100	c.T1025C	p.M342T	0.00077	NA	Tolerated	Probably damaging	No
FH	*CPT2*	4	rs17848485	c.A1634C	p.E545A	0.0007	0.004	Tolerated	Probably damaging	No
FH	*CYP2D6*	1	NA	c.137_138insT	p.L46fs	NA	NA	Tolerated	Probably damaging	Yes
FH	*CYP2D6*	3	rs28371710	c.G463A	p.E155K	0.01207	NA	Tolerated	Probably damaging	No
FH	*CYP2D6*	5	rs35742686	c.775delA	p.R259fs	NA	0.02	Tolerated	Probably damaging	Yes
FH	*CYP2D6*	7	rs202102799	c.A1064G	p.Y355C	NA	NA	Damaging	Probably damaging	No
FH	*GCKR*	19	rs34792470	c.C1768T	p.H590Y	0.00162	NA	Tolerated	Probably damaging	No
FH	*HNF1A*	1	rs1800574	c.C293T	p.A98V	0.018581	0.04	Tolerated	Benign	Yes
FH	*HNF1B*	1	NA	c.C148T	p.P50S	NA	NA	Tolerated	Probably damaging	No
FH	*HNF4A*	5	rs1800961	c.C395T	p.T132I	0.02376	0.03	Tolerated	Benign	Yes
FH	*LCAT*	5	NA	c.C746T	p.S249L	NA	NA	Tolerated	Probably damaging	No
FH	*LCAT*	5	rs4986970	c.T694A	p.S232T	0.022468	0.03	Tolerated	Possibly damaging	Yes
FH	*LCAT*	6	NA	c.1029delG	p.L343fs	NA	NA	Tolerated	Probably damaging	No
FH	*LDLR*	15	NA	c.C2177T	p.T726I	0.00577	0.0026	Tolerated	Benign	Yes
FH	*LIPC*	3	rs6078	c.G283A	p.V95M	0.04365	0.03	Tolerated	Benign	Yes

Supplemental Table 6 continued. Genetic variants identified in candidate gene mutation-free dyslipidemia patients

Class	Gene	Exon	rsID	Variant position		Variant frequency		*in silico* prediction		HGMD
				Nucleotide	Amino acid	ESP	G1K	SIFT	PolyPhen2	
FH	*LIPE*	1	rs141744211	c.C568A	p.P190T	0.004306	NA	Tolerated	Possibly damaging	No
FH	*LIPG*	8	rs77960347	c.A1187G	p.N396S	0.00977	0.01	Tolerated	Possibly damaging	Yes
FH	*LIPG*	10	NA	c.A1486G	p.T496A	NA	NA	Tolerated	Benign	No
FH	*LMF1*	7	rs143076454	c.C1060T	p.R354W	0.009556	0.03	Tolerated	Probably damaging	No
FH	*LMF1*	8	rs35168378	c.G1091A	p.R364Q	0.028904	0.03	Tolerated	Probably damaging	No
FH	*LMF1*	9	rs138205062	c.C1351T	p.R451W	0.003589	0.01	Damaging	Probably damaging	Yes
FH	*LMF1*	11	rs4984948	c.C1685G	p.P562R	0.00815	0.01	Tolerated	Probably damaging	No
FH	*LPIN1*	2	rs199755819	c.G61A	p.V21M	NA	NA	Tolerated	Probably damaging	No
FH	*LPL*	6	rs268	c.A953G	p.N318S	0.01338	0.02	Tolerated	Benign	Yes
FH	*MTTP*	4	rs2306986	c.G294C	p.E98D	0.089895	0.02	Tolerated	Benign	No
FH	*MTTP*	5	rs3792683	c.A497G	p.N166S	0.085653	0.02	Tolerated	Benign	No
FH	*MTTP*	7	rs17599091	c.C730G	p.Q244E	0.040674	0.03	Tolerated	Benign	No
FH	*MYLIP*	4	rs79992066	c.A604C	p.I202L	0.014609	0.02	Tolerated	Benign	No
FH	*NEUROD1*	2	rs8192556	c.C590A	p.P197H	0.019299	0.03	Damaging	Probably damaging	No
FH	*PAX4*	9	rs35434068	c.748-2->T	NA	NA	NA	Tolerated	Probably damaging	No
FH	*PCSK9*	1	rs11591147	c.G137T	p.R46L	0.009834	0.02	Tolerated	Benign	Yes
FH	*PLIN1*	7	rs74407840	c.C902T	p.T301M	0.00339	0.0026	Tolerated	Probably damaging	No
FH	*PNPLA2*	7	rs56152088	c.C793T	p.P265S	0.017589	0.02	Tolerated	Benign	Yes

Supplemental Table 6 continued. Genetic variants identified in candidate gene mutation-free dyslipidemia patients

Class	Gene	Exon	rsID	Variant position		Variant frequency		*in silico* prediction		HGMD
				Nucleotide	Amino acid	ESP	G1K	SIFT	PolyPhen2	
FH	*PYGM*	13	rs139570786	c.A1537G	p.I513V	0.002924	0.004	Tolerated	Benign	No
FH	*PYGM*	17	rs113806080	c.C2009T	p.A670V	0.003155	0.01	Damaging	Probably damaging	Yes
FH	*SCARB1*	3	rs5891	c.G403A	p.V135I	0.010226	0.01	Tolerated	Benign	Yes
FH	*SLC22A8*	10	rs11568486	c.G1342A	p.V448I	0.008386	0.01	Tolerated	Benign	No
FH	*SORT1*	3	rs61797119	c.A370G	p.I124V	0.004306	0.004	Tolerated	Benign	No
FH	*SORT1*	11	rs144141753	c.A1340G	p.E447G	0.001	0.0026	Tolerated	Benign	No
FH	*ZMPSTE24*	2	NA	c.138_140del	p.46_47del	NA	NA	Tolerated	Probably damaging	No
FH, HTG	*ABCA1*	16	rs2066718	c.G2311A	p.V771M	0.054975	0.02	Tolerated	Benign	Yes
FH, HTG	*APOA4*	1	rs12721041	c.G37A	p.V13M	0.012013	0.01	Damaging	Possibly damaging	No
FH, HTG	*LPIN1*	12	rs33997857	c.G1735A	p.V579M	0.0163	0.02	Damaging	Benign	No
FPLD	*ABCG5*	11	rs140899003	c.A1567G	p.I523V	0.001845	NA	Tolerated	Benign	No
FPLD	*NPC1L1*	2	NA	c.G112A	p.E38K	0.000077	NA	Tolerated	Possibly Damaging	No
HTG	*ANGPTL3*	4	rs77871363	c.T776C	p.M259T	0.0163	NA	Tolerated	Benign	No
HTG	*BLK*	5	rs115068920	c.T335C	p.F112S	0.00684	NA	Damaging	Probably damaging	No
HTG	*CETP*	1	rs34065661	c.C44G	p.A15G	0.02255	NA	Tolerated	Benign	No
HTG	*CREB3L3*	3	rs145839480	c.G334A	p.G112S	0.00354	NA	Tolerated	Benign	No
HTG	*LIPG*	9	rs117623631	c.C1426T	p.R476W	0.00177	0.0026	Damaging	Possibly damaging	Yes
HTG	*LPL*	2	rs1801177	c.G106A	p.D36N	0.02706	0.01	NA	Possibly damaging	Yes

**Supplemental Table 6 continued. Genetic variants identified in candidate gene mutation-free dyslipidemia patients**										
				**Variant position**		**Variant frequency**		***in silico* prediction**		
**Class**	**Gene**	**Exon**	**rsID**	**Nucleotide**	**Amino acid**	**ESP**	**G1K**	**SIFT**	**PolyPhen2**	**HGMD**
HTG	*PAX4*	2	rs112061448	c.G187T	p.G63C	0.002	NA	Damaging	Benign	No
HTG	*PLIN1*	7	rs58361219	c.C812T	p.A271V	0.00612	NA	Tolerated	Benign	No
HTG	*PTRF*	2	NA	c.C847T	p.P283S	0.00023	NA	Tolerated	Benign	No
HTG	*PYGM*	10	rs71581787	c.C1184T	p.T395M	0.01818	0.0013	Damaging	Benign	No
HTG	*SCARB1*	12	rs701103	c.G1495A	p.G499R	0.05349	0.0013	Tolerated	NA	No
HTG, FPLD	*CYP2D6*	7	rs150552908	c.G1117A	p.G373S	NA	0.02	Tolerated	Benign	No
MODY	*APOB*	26	rs72653095	c.C8462T	p.P2821L	0.00262	0.01	Tolerated	Possibly damaging	Yes
MODY	*MTTP*	14	NA	c.A1811G	p.N604S	NA	NA	Tolerated	Probably damaging	No

Abbreviations as in Supplemental Tables 1-4
